# Molecular Immune Targeted Imaging of Tumor Microenvironment

**DOI:** 10.7150/ntno.66556

**Published:** 2022-02-15

**Authors:** Taha Rakhshandehroo, Bryan Ronain Smith, Hannah J. Glockner, Mohammad Rashidian, Neeta Pandit-Taskar

**Affiliations:** 1 Department of Imaging, Dana-Farber Cancer Institute, Harvard Medical School, Boston, MA, USA; 2 Department of Biomedical Engineering, Michigan State University, East Lansing, MI, USA; 3 Department of Radiology, Memorial Sloan Kettering Cancer Center, New York, NY and Weill Cornell Medical College, New York, NY, USA

**Keywords:** Biomarker, radiolabeled antibody, checkpoint inhibitors, PD-1, PD-L1, FDG

## Abstract

Novel targeted therapies are rapidly emerging for the treatment of cancer. With the advent of new immune targeting agents, understanding the changes in the tumor microenvironment (TME) is critical. Given the complexity and several cellular mechanisms and factors that play a role in the TME, novel imaging methods to assess and evaluate the dynamic changes in the TME during treatment are needed. Several techniques are being developed for imaging TME including optical, fluorescence and photoacoustic methods. Positron emission tomography (PET) imaging can be used to track the dynamics of different molecular targets in the TME in live animals and in humans. Several novel PET imaging probes including radiolabeled antibodies, antibody fragments, and small molecules have been developed with many more that are under development preclinically and in early human studies. This review is a brief overview of some of the PET agents that are either in the preclinical developmental phase or undergoing early clinical studies.

## Introduction

Immune-directed therapies of malignancies are a growing strategy for treatment of multiple tumors. Current treatment strategies face challenges in clinical management, mainly related to variable responses and outcomes across different malignancies and patients. Several physiological factors and processes of the tumor microenvironment such as changes in extracellular matrix components, vascular or lymphatic structures in tumor, cellular alterations, stromal cell derived proteases or physiologic processes such as hypoxia, interstitial pressure and pH play a complex role in tumor growth and response to treatment 1,2. Assessment of tumor microenvironment (TME) is critical for prognostication and optimizing the choice of targeting agent and dynamic evaluation of TME can help assess treatment effectiveness 3.

Noninvasive imaging plays a major role in the management of patients with cancer. Several imaging methods are currently being clinically utilized that provide unique information and allow for imaging multiple processes in tumor microenvironment. These broadly include imaging modalities such as optical imaging, functional imaging with positron emission tomography (PET) or single-photon emission tomography (SPECT), magnetic resonance imaging/spectroscopy (MRI/MRS), and ultrasound imaging. An imaging modality that accurately provides predictive and prognostic information is lacking and there is a critical unmet need for novel prognostic imaging agents. Additionally, given the complexity of the TME, monitoring dynamic changes during treatment and predicting outcomes is a challenge. Functional PET imaging with ^18^F-fluorodeoxyglucose (^18^F-FDG) has proven useful not only as a sensitive tool to detect and stage cancer, but also as a biomarker for prediction and prognostication in the treatment of malignancies and is currently the most widely used PET imaging radiotracer for management of malignancies 4. However, ^18^F-FDG is an analog of glucose and is taken up by metabolically active glucose-avid cells expressing the GLUT1 and/or GLUT3 transporters 5. Thus, besides cancer cells, activated immune cells in tumor or those associated with inflammation and physiologically glucose-avid tissues such as the brain and heart as well as inflammatory cells can thus uptake ^18^F-FDG. ^18^F-FDG therefore is not specific and has no ability to delineate subpopulations of cells, thereby limiting its power to evaluate TME and outcomes for immune-directed therapies. Novel targeted imaging agents are needed in order to detect and differentiate subclasses of cells within the TME.

Several antibody-based therapies exploiting tumor, immune, or vascular markers have been developed as cancer therapeutics. Such targeted agents can be useful to noninvasively monitor the composition of the TME, using functional imaging. Preclinical and early clinical studies to image subpopulations of immune cells, vasculature, presence or absence of cytokines, and to determine the suitability of a therapeutic strategy such as bi-specifics or CAR-T cell therapies are being conducted. Several imaging approaches generally utilize targeting agents such as antibody, antibody fragment, peptide or ligand, or other small molecules such as aptamer. Other techniques include reporter molecules that can be targeted for functional imaging using nanoparticles, fluorophores, or radionuclides. While several imaging techniques are being used, this review focuses primarily on functional imaging using radioisotopes and includes a brief discussion on nanoparticles.

### Imaging using radiolabeled small molecules or antibodies

Small molecules, such as radiolabeled sugars, hormones, drugs, and nucleosides are the most prevalent form of radiolabeled agents for PET imaging. An advantageous attribute of these small molecules is the ability to permeate cell membranes and, therefore, access targets intracellularly. Additionally, due to their small size, these tracers rapidly clear from circulation and have higher tissue penetrance and permeation compared to antibody-based tracers.

Despite the favorable attributes of small molecules as imaging agents, they pose challenges as imaging agents to assess the immune response to cancer. Due to rapid metabolism and excretion, there can be high uptake in the kidneys and bladder.

Antibody-based PET imaging platforms allow for highly specific targeted imaging of immune processes. As compared to small molecules, the antibodies are larger molecules (~150 kDa) that may limit tissue penetration; the Fc region of antibodies binds to neonatal Fc receptor (FcRn), recycling the antibody back into circulation and protecting it from lysosomal degradation that also results in longer circulation. However, the advantage of antibodies is that they best resemble the characteristics of an antibody-drug. Smaller antibody fragments such as minibodies, cys-diabodies, single-chain Fvs (scFvs), and nanobodies have been developed and used for PET imaging. Antibody-fragments as imaging agents have combined advantage of smaller size resulting in more rapid clearance and higher tissue penetration as compared to antibodies.

## Imaging immune response

In order to target immune cells and differentiate them from cancer cells, small molecule radiotracers have been developed targeting specific metabolic pathways that are upregulated in activated immune cells (Table 1). Most tissues rely on the *de novo* DNA synthesis pathway to produce nucleotides to support cellular growth and division. However, rapidly expanding immune cells utilize the nucleoside salvage pathway for DNA synthesis 6. Deoxycytidine kinase (dCK) is highly expressed in lymphocytes and is the rate-limiting enzyme in the nucleoside salvage pathway 7. 1-(2'-deoxy-2'-[^18^F]fluoroarabinofuranosyl) cytosine (^18^F-FAC), a nucleoside analog that is specific towards dCK, shows specific labeling of proliferating CD8^+^ T cells 8. Preclinical studies indicated high signal-to-noise in lymphoid organs and bone marrow while *ex vivo* analysis illustrated high uptake in T cells, B cells, and CD11b^+^ myeloid cells. However ^18^F-FAC is likely not a suitable imaging tool for ultimate clinical translation because it is rapidly catabolized by cytidine deaminase 9.

**Figure 1:**
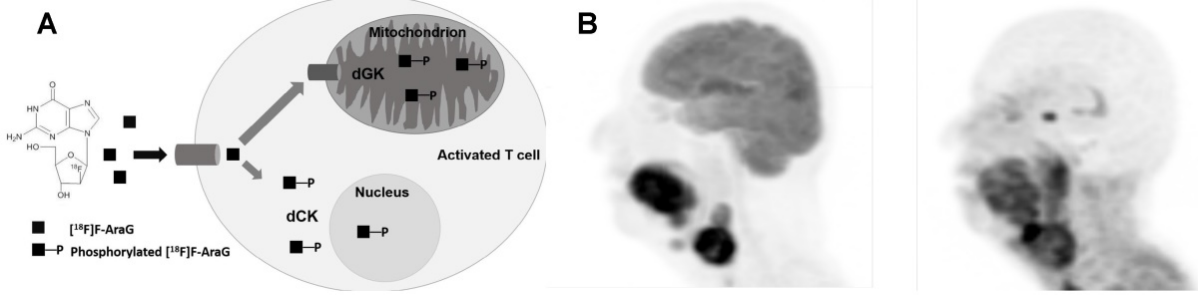
**(A)** Mechanism of uptake of ^18^F-ARAG: Reproduced from Levi J et al. CCR (doi:10.1158/0008-5472.CAN-19-0267). **(B)** Patient with head and neck carcinoma. PET/CT imaging with ^18^F-FDG and ^18^F-ARAG shows prominent uptake in the lesions in larynx; uptake is also noted in ^18^F-ARAG scan with heterogenous uptake. (Courtesy: Drs. Colevas, Sunwoo, and Davidzon, Stanford University) (Reprinted with permission from Elsevier, Pandit-Taskar N, Postow MA. Immune-Directed Molecular Imaging Biomarkers. Semin Nucl Med. 2020 Nov;50(6):584-603. doi: 10.1053/j.semnuclmed.2020.06.005. Epub 2020 Jul 15. PMID: 33059826.)

To overcome this issue, 9-(β-D-Arabinofuranosyl) guanine (AraG), a non-catabolizible imaging agent, was developed. As a guanosine analog, it can be phosphorylated by dCK and, consequently, trapped intracellularly in T cells *in vivo* without any toxicity 10, 11, therefore serving as an ideal targeted small molecule PET imaging tracer (Figure 1). In a murine model of colorectal cancer, two days post-administration of anti-PD-1 treatment, responder mice showed a significantly higher ^18^F-AraG PET signal in their tumors relative to a non-responder mice, indicating ^18^F-AraG PET was able to successfully predict the response to programmed death-1 (PD-1) blockade 11. In patients, ^18^F-AraG preferentially accumulates in activated CD8^+^ T cells with about 7 times higher uptake in activated T cells as compared to murine cells 10. Currently, four phase I (NCT04524195, NCT04052412, NCT04678440, NCT04186988) and two phase II (NCT04726215, NCT04260256) human studies are underway to study biodistribution and kinetics in patients with non-small cell lung cancer (NSCLC) treated with checkpoint blockade.

Although ^18^F-AraG can target expanding immune cells, it is not a representative readout for regions of T cell activity and killing. Targeted small molecules that bind to markers of T cell activity, either as released molecules or expressed on the cell surface, can be used to assess the T cell response to cancer. Granzyme B is a serine protease found in the granules of cytotoxic T cells and has been recognized as an important downstream effector of tumoral cytotoxic T cells. Granzyme B, upon secretion into the target cell through perforin-mediated pores, can cleave and activate caspase 8 and 10, leading to a signaling cascade that triggers apoptosis of the targe cell. Therefore, imaging granzyme B is expected to distinguish an active immune response from exhausted T cells. Granzyme B also stimulates the release of cytokines, can induce inflammation, and also is involved in extracellular matrix remodeling. Therefore, it has gained interest as a potential biomarker to predict the efficacy of immunotherapies 12. A ^68^Ga-radiolabeled granzyme B-binding peptide (GZP) has been designed from the cleavage sequence of murine granzyme B, which has been modified to promote irreversible binding 13. In a colorectal preclinical cancer model, tumors that responded to anti-CTLA-4 and anti-PD-1 treatment and imaged 1 hour post-injection of the tracer showed high uptake in the tumor while non-responders showed low tumor uptake 13. Furthermore, in tumor models treated with a combination of checkpoint inhibitors, ^68^Ga-NOTA-GZP tracer uptake in the treatment group linearly correlated with percent response across all therapies, demonstrating its value as a predictive marker 14. A first in-human phase I clinical imaging study (NCT04169321) to assess safety and kinetic of ^68^Ga-NOTA-hGZP is being conducted in 20 patients with melanoma or non-small cell lung cancer treated with pembrolizumab.

Antibodies or fragments have been developed to image myeloid cells that play key roles in shaping the immune status of the TME 15-17, 18. Imaging their presence and activation status can help to better assess the response to immunotherapy. Tumor-associated macrophages can significantly influence the TME immune landscape by secreting different cytokines. M1-like macrophages secrete inflammatory cytokines, such as CCL5, CXCL9, and CXCL10, which can recruit and activate T cells, whereas M2-like macrophages secrete cytokines that repel T cells. Thus, imaging the presence and phenotypic status of macrophages in the TME can provide valuable information for the assessment of immunotherapy response. A ^99m^Tc-labeled nanobody targeting the macrophage mannose receptor (MMR, CD206), a marker highly expressed by the immunosuppressive M2-like macrophages, has been utilized for SPECT imaging in preclinical models of lung and mammary tumors. This imaging agent was able to detect MMR^+^ macrophages in hypoxic regions of the tumor with clarity three hours post-injection 18. Further preclinical PET imaging studies utilizing a human/mouse MMR cross-reactive ^18^F-labeled nanobody in a mouse model of lung cancer validated MMR^+^-macrophage imaging, suggesting it can serve as a suitable imaging tool for ultimate clinical translation 19.

CD11b is a myeloid cell marker and has served as a target for PET imaging of myeloid cells. An anti-CD11b ^99m^Tc-labelled antibody was used in a mouse colon cancer model and showed high uptake in tumors six hours post-injection, indicating the presence of myeloid cells 20. Similarly, an anti-CD11b nanobody was used to image tumor infiltration of myeloid cells in a melanoma model and could detect the tumor with clarity. Interestingly, penetration of CD11b^+^ cells to the tumor core was not found to be a negative predictor of tumor response to PD-1 blockade 21, potentially due to the diversity and polarization of CD11b^+^ cells, which can serve as both anti-tumor and pro-tumor roles.

Professional antigen presenting cells, including macrophages, B cells, and dendritic cells, are pivotal to development of an anti-tumor immune response. These cells are unique in their expression of MHC-II, which is used to present tumor antigens to CD4^+^ T cells. A nanobody against mouse MHC-II was developed and used for *in vivo* detection of tumor infiltrating MHC-II^+^ cells in both syngeneic and xenogeneic preclinical models. The ^18^F-radiolabaled nanobody could detect MHC-II^+^ cells in the tumor two hours post-injection 21. An anti-human MHC-II nanobody was developed and used to image MHC-II^+^ cells in a humanized mouse model of graft versus host disease (GvHD). Mice that developed GvHD showed high uptake of the anti-MHC-II tracer in the liver 22. Performing single-cell RNA sequencing on tumor infiltrating immune cells obtained from a responder and a non-responder mouse treated with PD-1 blockade showed a significant increase in the expression profile of class II MHC molecules on tumor immune infiltrating cells in the responder animal 21, suggesting that imaging class II MHC can be used to evaluate a response to treatment. The higher expression is potentially due to higher presence of IFN-γ in the TME of the responding tumor. The ability to predict and prognosticate using this target is yet to be proven. Additionally, the lack of ability to distinguish between different APCs using MHC-II to target can be limiting.

As innate lymphoid cells, natural killer (NK) cells interact with and destroy tumors via different mechanisms than T cells. NK cell activation can represent anti-tumor activity. Antibodies to NKp30, an activation natural cytotoxicity receptor expressed on NK cells, were radiolabeled with ^64^Cu or ^89^Zr. These stable and specific radiolabeled antibodies were injected into several murine xenograft and adoptive cell transfer models of renal cell carcinoma and imaged via PET to visualize NK cell activation.

## Imaging checkpoint molecules

### Preclinical imaging

**Figure 2:**
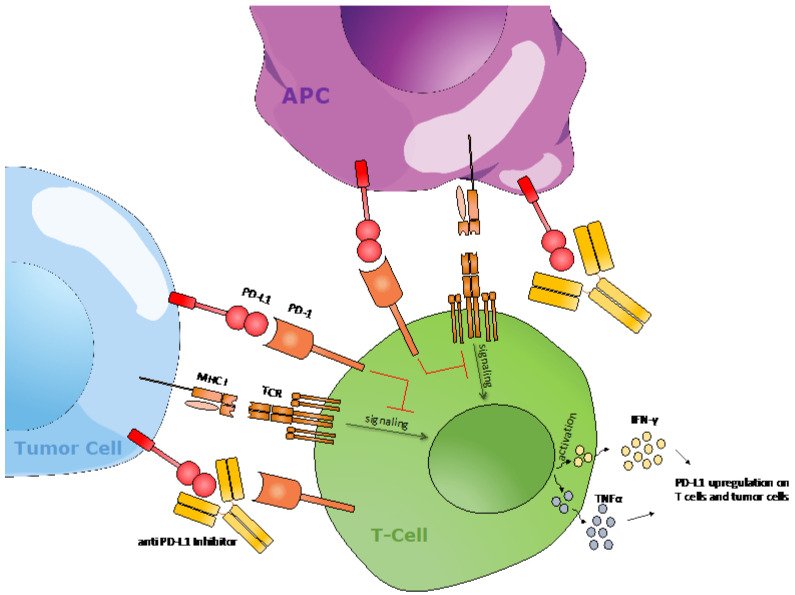
PD-1/PD-L1 checkpoint molecules regulate T cell activation. Upon peptide-MHC interaction with its T cell receptor in concurrence with costimulatory signals, T cells are activated, resulting in T cell-mediated killing and the release of cytokines, such as IFN-γ and TNFα. The interaction between PD-1 and PD-L1 results in the downregulation of anti-tumor T cell signaling. An agonistic anti-PD-(L)1 antibody will disrupt this interaction and subsequently help with T cell activation.

Checkpoint blockade treatments, such as anti-PD-1 and anti-CTLA-4 have shown impressive success in the treatment of several malignancies such as melanoma and non-small cell lung cancer (NSCLC). Checkpoint inhibitor therapies act by preventing immune suppression via temporarily blocking the interaction of checkpoint molecules with their receptors, resulting in continued activation of T cells that can lead to a more efficient anti-tumor immune response. Several studies have shown expression of PD-L1 is correlated to the response to PD-1 blockade (Figure 2) 23. However, currently, the only clinically viable method to assess tumor expression of PD-L1 protein is through tumor biopsies, which is invasive, susceptible to sampling errors, practically restrictive, and lacks the ability to capture the heterogeneity of a tumor. Therefore, several approaches have been developed to noninvasively image PD-L1.

A cyclic 14-residue peptide, named WL12, that binds to PD-L1 with low nanomolar affinity (IC50 ~ 26-32 nM) was developed and used for PET imaging of PD-L1 expression. In mice bearing cancer xenografts, ^18^F-WL12 showed high specificity and affinity for PD-L1. The specific uptake of ^18^F-WL12 in tumors was shown to be reduced when cold peptide was injected prior to imaging, further showing the specificity of the observed signal. The biodistribution analysis of ^18^F-WL12 showed prominent uptake in hepatic, renal, and normal tissues. High tumor uptake was observed 2 hours post injection with %ID/g ranging from 7.16-8.86 at 1 and 2 hours post-injection 24. A phase I clinical study in patients with solid tumors is planned (NCT04304066).

**Figure 3:**
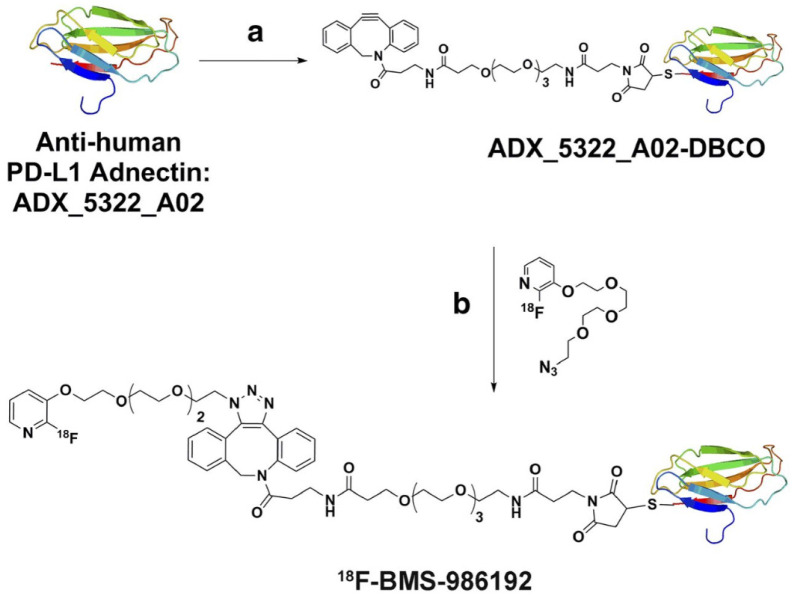
Antihuman PD-L1 adnectin. Structure and synthesis of ^18^ F-BMS986192 antiPD-L1 adnectin. (Adapted from a figure that was originally published in JNM. Donnelly D et al. Synthesis and Biologic Evaluation of a Novel ^18^ F-Labeled Adnectin as a PET Radioligand for Imaging PD-L1 Expression. J Nucl Med. 2018;59(3):529-535. © SNMMI.)

**Figure 4:**
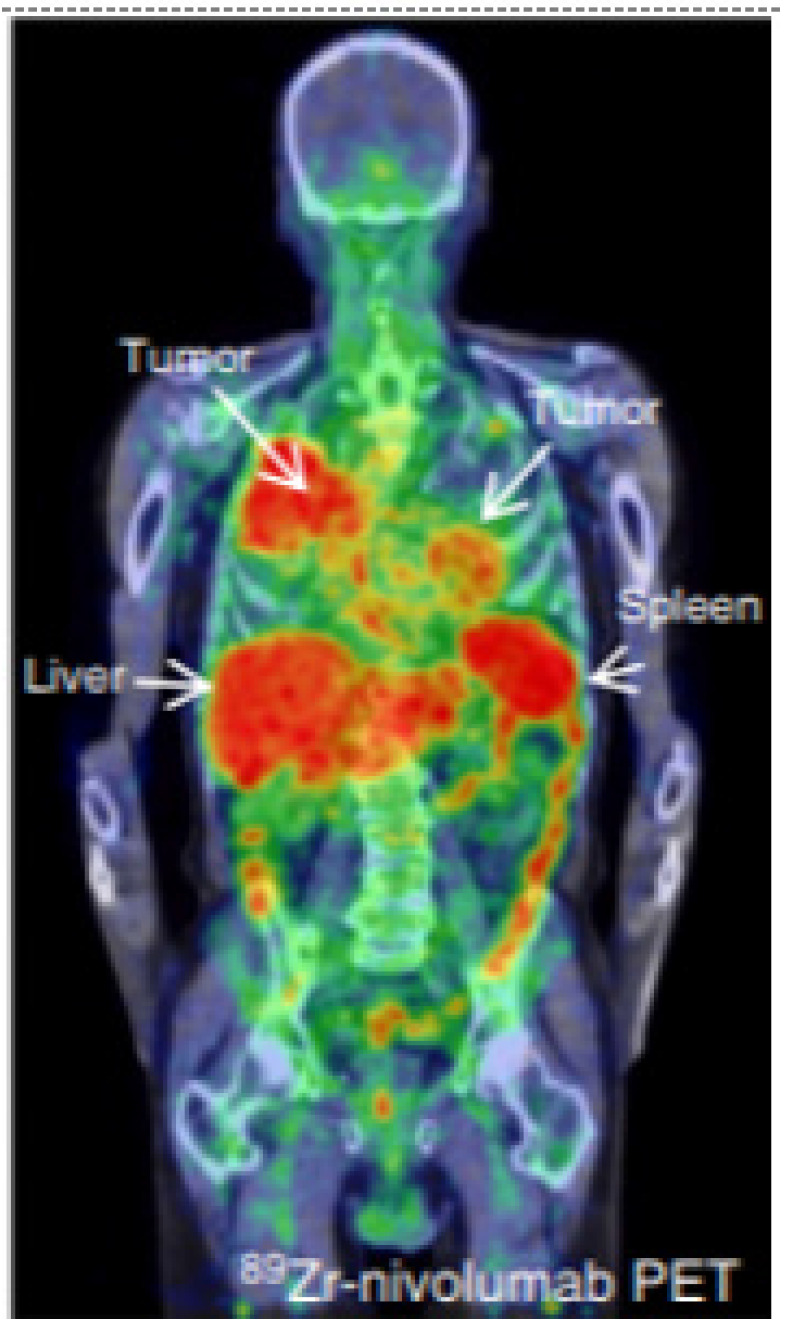
^89^Zr-labeled Nivolumab PET (37.09 MBq, 162 h p.i.) demonstrate heterogeneous tracer uptake within and between tumors. (Adapted for use under Creative Commons license http://creativecommons.org/licenses/by/4.0/ from Niemeijer, A.N., Leung, D., Huisman, M.C. *et al.* Whole body PD-1 and PD-L1 positron emission tomography in patients with non-small-cell lung cancer. *Nat Commun* **9,** 4664 (2018). https://doi.org/10.1038/s41467-018-07131-y)

Adnectins, small protein molecules of ~10kDa size, have been developed against different targets as imaging agents. Adnectins are engineered based on the framework of the human 10th fibronectin type III domain (^10^FN3). They are structurally similar to variable domains of antibodies and are suitable as PET imaging agents owing to their small size, a non- or low-immunogenic profile, and pharmacokinetic properties 25. Adnectins lack disulfide bonds and can be designed against a specific target with low nanomolar or even picomolar affinities. ^18^F-BMS-986192, an adnectin specific for PD-L1, was developed and used for imaging PD-L1 in lung cancer patients (Figure 3). The biodistribution showed uptake in marrow and spleen, sites of high PD-L1 expression, while uptake in lesions showed heterogeneity between patients as well as for different lesions in a patient. ^89^Zr-Nivolumab is radiolabeled an anti-PD-1 antibody that has shown ability to assess target expression in patients (Figure 4). Combined imaging with ^89^Zr-Nivolumab and ^18^F-BMS-986192 allowed for simultaneous assessment of both PD-1 and PD-L1, that demonstrated variation in uptake, thereby expression of PD-1 and PD-L1 across lesions in patients 26, 27. Preclinical imaging in mice noted 3.5 times higher uptake in tumors expressing PD-L1 as compared to control animals. Specificity was further confirmed with blocking of radioligand binding in a dose-dependent manner 28.

### Clinical imaging

**Figure 5:**
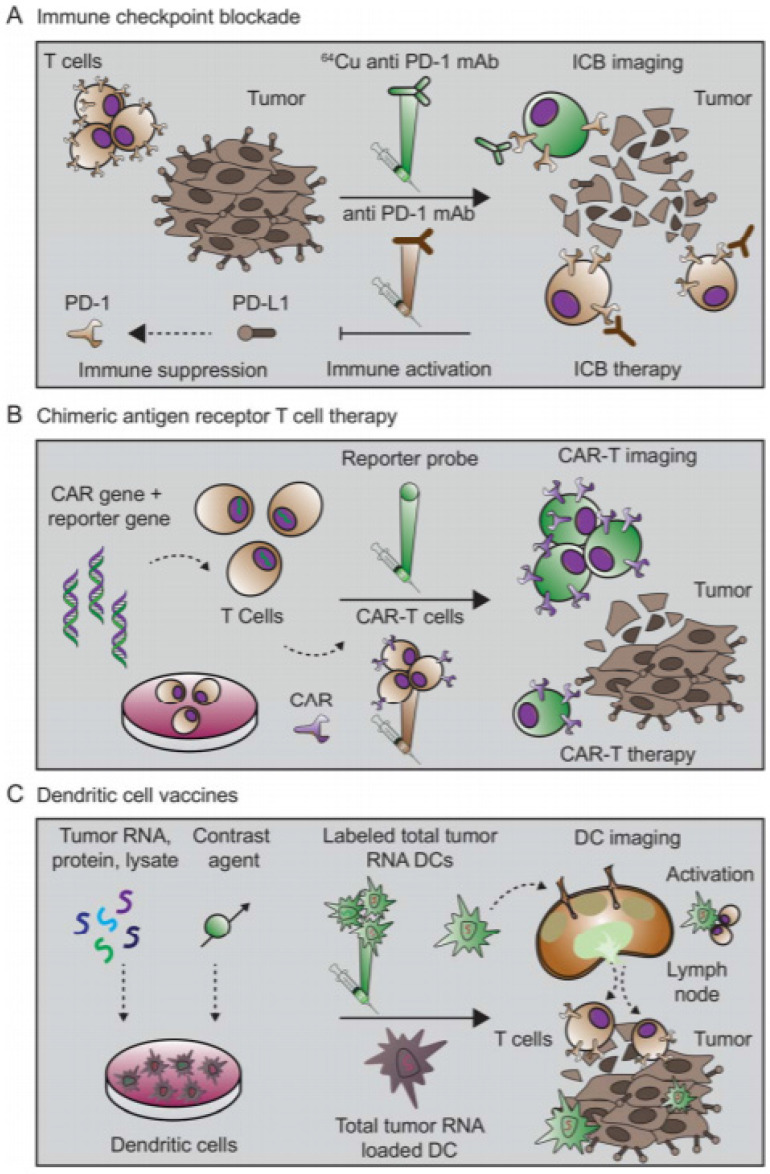
Immuno-imaging applications toward immunotherapy. Immune checkpoint blockade, CAR-Ts, and therapeutic vaccines constitute three important classes of cancer immunotherapy. **(A)** Immune checkpoints that regulate anti-tumor immunity have now been identified as promising therapeutic targets. Blocking signaling pathways that suppress anti-tumor immune responses has proven especially effective. In one approach, an anti-PD-1 mAb targets the PD-1 receptor on T cells, blocking ligation of the receptor and immunosuppression by PD-L1 on tumor cells. Anti-PD1 mAb administration thus leads to immune activation and therapeutic response. Imaging the expression of PD1 with a radiolabeled mAb may assist with selection of patients for treatment, optimal dosing, and response monitoring. **(B)** CAR-T strategies engineer a patient's immune cells *ex vivo* to express a receptor that can bind specifically to tumor cells. During this engineering process, a reporter gene can also be inserted to enable longitudinal tracking of the CAR-T cells. Upon administration, CAR-Ts seek out and destroy malignant tumor cells. Subsequent imaging with a reporter probe can give insights into their location and functional status. **(C)** Cancer vaccine strategies come in many formulations. In one approach, dendritic cells are pulsed with tumor antigen, lysate, or RNA. Dendritic cells then express tumor antigens on their MHC molecules, which are capable of eliciting a T cell-driven immune response. Successful responses require homing of dendritic cells to the lymph nodes and tumor. At these sites, the dendritic cells are capable of activating tumor-specific T cells. Labeling the dendritic cells with a contrast agent allows for assessment of successful homing of dendritic cells to lymph nodes and other secondary lymphoid sites. This knowledge can be utilized to inform both dose and route of vaccine administration. (Adapted from a figure that was originally published in* JNM*. Mayer AT and Gambhir SS. The Immunoimaging Toolbox. J Nucl Med. 2018;59(8):1174-1182. © SNMMI.)

Immune checkpoint inhibitor therapy is rapidly becoming the mainstay in the management of several malignancies. Over the last several years, many radiolabeled antibodies targeting the checkpoint molecules have been developed and some are already in early clinical assessment (Figure 5).

**Figure 6:**
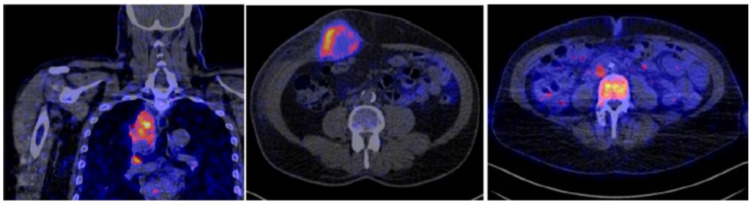
Radiolabeled ^89^Zr-atezolizumab imaging in patients showing heterogenous uptake within lesions in the thorax: (left) mediastinal lesion in a patient with lung cancer, (middle) abdominal wall metastatic bladder cancer, and (right) node in abdomen and metastatic bone lesion from breast cancer (arrows). (Adapted from Bensch E et al. Nat Med. 2018 Dec;24(12):1852-1858.)

Pembrolizumab and Nivolumab, FDA-approved monoclonal (IgG4) antibodies for targeting PD-1 receptors, have been radiolabeled with ^64^Cu or ^89^Zr for imaging (Table 2). Preclinical assessment with ^89^Zr-Pembrolizumab showed specific targeting of T cells in the spleen and in salivary and lacrimal glands, confirmed by tissue staining. The retained activity in tissue was long, with a liver clearance half-life of 168 hours 29. Distribution of ^89^Zr-Nivolumab in healthy primates showed similar distribution with prominent uptake in liver, spleen, and lymph nodes, sites rich in T cell infiltration, confirmed with blocking of uptake with cold Nivolumab and histology analyses 30, 31. Low uptake was noted in other tissues such as brain, heart, lung, kidney, and muscle, while the nodal uptake increased with time, suggesting continuous binding to T cell and accumulation. Atezolizumab and Durvalumab antibodies target PD-L1 and are FDA-approved for the treatment of certain malignancies, either as a single agent or in combination with other agents. Imaging with ^89^Zr-atezolizumab in 22 patients with either NSCLC, triple-negative breast cancer, or bladder cancer showed good targeting of tissues and lesions with PD-L1 expression, as correlated with immunohistochemistry 32. There was prominent splenic, nodal, and bone marrow uptake and correlation of uptake to CD8 expression was noted in spleen though some uptake was also noted in some sites of inflammation. Tumor uptake was heterogenous and varied between tumor types and sites of lesions (Figure 6). Lower uptake was noted in breast cancer lesions compared to bladder or lung cancer and in bone lesions compared to liver lesions. Higher ^89^Zr-atezolizumab uptake in lesions was noted in those with better response and ^89^Zr-atezolizumab uptake showed better correlation than with immunohistochemistry or RNA-sequencing-based predictive biomarkers 32. ^89^Zr-atezolizumab imaging may be useful to stratify and identify patients with RCC who are most likely to benefit from PD-1/PD-L1-directed treatment 33. ^89^Zr-Durvalumab imaging trials in head and neck cancer and NSCLC undergoing treatment with durvalumab are underway 34 (NCT03853187 and NCT03829007).

Lymphocyte-activation gene 3 (LAG-3) is a cell surface marker predominantly expressed in CD3-positive T cells with low expression in normal or malignant B cells, NK cells, and myeloid cells 35 and in PD-1-positive CD8^+^ and CD4^+^ tumor-infiltrating lymphocytes. LAG-3 negatively regulates T cell proliferation and activation with a role in suppressing the active T cells 36. ^89^Zr-REGN3767 is a radiolabeled antibody against LAG-3 antibody that is being evaluated in a first-in-human study in patients with lymphoma (NCT04566978) and other advanced malignancies (NCT03005782).

## Imaging engineered T cells

### Preclinical imaging

**Figure 7:**
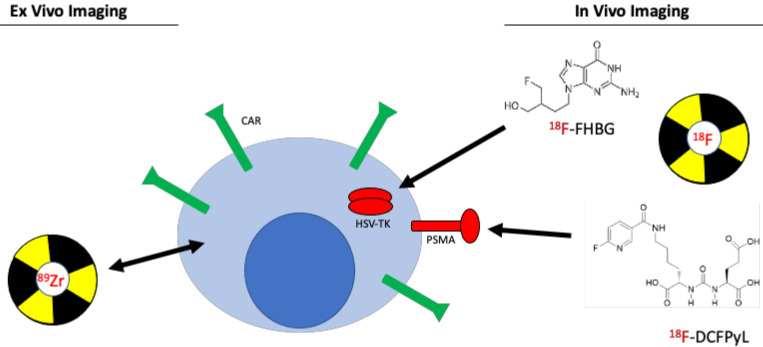
Imaging chimeric antigen receptor (CAR) T cells. Two different approaches are commonly used to image engineered T cells. *Ex vivo* labeling involves direct use of the PET radiotracer on the engineered T cell, either through binding of the tracer to proteins on the plasma membrane or through passive transport across the plasma membrane. This method is simple and easy to conduct, however, due to effluxion over time as well as dilution due to T cell expansion, same-day imaging is required. *In vivo* labeling is a much more robust and utilized imaging method. The method utilizes a small molecule PET radiotracer specific to a reporter gene that is co-expressed with the chimeric antigen receptor. This approach allows for longitudinal assessment of CAR T cells.

Chimeric antigen receptor (CAR) T cells have shown great promise in treatment of blood-borne malignancies. CAR is a synthetic receptor capable of binding to a target and eliciting an activation and expansion signal in T cells such that it, ultimately, causes the killing of the target cell (Figure 7). Currently, CAR T cells specific for CD19 have been FDA-approved for the treatment of various B cell malignancies such as large B cell lymphoma or acute lymphoblastic leukemia (ALL) 37, 38, while BCMA CAR T cells were recently approved for the treatment of multiple myeloma 39. Despite these encouraging results, patients face detrimental side effects, such as cytokine release syndrome 40 or B cell aplasia 41, 42. Therefore, visualizing and imaging the behavior and dynamics of CAR T cells *in vivo* can become a robust clinical tool to assess or even predict response to CAR T cell treatment, for which several strategies have been developed (Table 3).

Direct *ex vivo* labeling of immune cells isolated from patients with a radioactive tracer after being transduced and just before being adoptively transferred allows the CAR T cells to be monitored longitudinally by PET imaging over time *in vivo*. This strategy enables simple, rapid, and specific labeling. However, the first instance of *ex vivo* labeling of human immune cells with ^99m^Tc found only 50% cellular viability post-labeling 43. Therefore, ionophore chelators, such as 8-hydroxyquinoline (oxine) or hexamethylpropyleneamine (HMPAO), which carry radioisotopes across the plasma membrane, were developed to increase labeling efficiency and viability. Utilizing these ionophores revealed 98% viability with 73% and 44% labeling efficiency when using ^111^In-oxine and ^99m^Tc-HMPAO, respectively 44.

A preclinical study used ^89^Zr-oxine to label anti-IL-13R2 CAR T cells and found labeling efficiency was 75% while labeled cells retained more than 60% of the ^89^Zr after 6 days *in vitro* 45. The labeled CAR T cells were delivered intraventricularly to mice bearing patient-derived glioblastoma multiforme (GBM) cancer cells. The CAR T cells were detectable by PET at least 6 days post-injection within intracranial tumors with no effect on CAR T cell-mediated tumor killing 45. These results indicate that *ex vivo* labeling maintains CAR T cell function and PET tracer signal *in vivo* throughout a relevant timeframe.

*Ex vivo* labeling is a simple technique to label immune cells. Because CAR T cells are transduced and expanded *ex vivo*, the *ex vivo* labeling approach can be a simple addition to a clinical protocol. Although, upon infusion, the expansion of CAR T cells will dilute the radiotracer among newly expanded cells causing a decrease in signal. Therefore, imaging must be conducted in a short timeframe after infusion. Furthermore, decay and effluxion of the radioisotope also necessitate immediate PET imaging upon CAR T cell infusion. Therefore, other methods, such as *in vivo* imaging, should be utilized that require less restricting imaging needs. In particular, while long-term cell labeling typically requires introduction of a reporter gene, shorter-term labeling can be accomplished by *in vivo* immune cell labeling or *ex vivo* cell labeling and re-injection 46.

In order to overcome the shortcomings of *ex vivo* labeling, imaging CAR T cells using a reporter gene can be used. A reporter gene expressed in CAR T cells allows for imaging *in vivo* using a radioactive small molecule specific for the reporter. This strategy allows for imaging despite migration, homing, and expansion of the CAR T cell, making imaging of cell trafficking at any time point possible *in vivo*. Herpes simplex virus thymidine kinase (HSV-TK), human norepinephrine transporter (hNET), and sodium-iodide symporter (hNIS), among others, are established reporter genes that have been used to image CAR T cells 47. HSV-TK, however, can serve not only as a PET reporter imaging system, but also in a bifunctional role a potential suicide switch for CAR-T cells upon infusion of a drug such as ganciclovir 48.

The HSV-TK has been widely used as a PET reporter gene system. Upon infusion of the CAR T cells expressing HSV-TK, ^18^F-labeled Penciclovir, a small molecule inhibitor for HSV-TK, can be administered, which will target the CAR T cells. The advantage of this reporter gene is the specificity of the small molecule and the ability to inject the targeted imaging agent at any timepoint. This system shows high specificity and low background, especially when using a mutant HSV-TK with higher affinity for Penciclovir. However, a concern that exists with the HSV-TK reporter system is a potential immune response against the reporter. Therefore, human reporter genes, such as sodium iodide symporter (hNIS) 49, norepinephrine transporter (hNET) 50, and somatostatin receptor 2 (SSTR2) 51 have been used to label CAR T cells. Each of these human reporter systems have certain advantages and disadvantages. For example, the advantage of hNIS is that it is non-immunogenic, not internalized, and is not expressed in necrotic cells 52. However, the hNIS reporter system has high background because hNIS is expressed in many normal epithelial tissues and carcinomas 53. In contrast, SSTR2 human reporter gene has an advantage because of its low baseline expression in normal tissue as well as available clinically approved specific radiotracers, such as ^68^Ga octreotide analogues (^68^Ga-DOTATOC) 54. However, SSTR2 is not as sensitive due to internalization upon interaction with ligands 55.

PET imaging employing split reporter strategies has also been used to evaluate protein-protein interactions *in vivo* based on protein-fragment complementarity. The basis of this approach is that splitting a reporter protein into two fragments abolishes function, but when the fragments are brought within close proximity, reporter activity is partially restored 56. When parts of the reporter protein are split between two interacting proteins, the dynamic localization of their interactions can thus be visualized. Using HSV-TK, PET imaging can visualize the interactions. An early example of this approach visualized protein-protein interactions of several protein partners, such as hypoxia-inducible factor-1α (HIF-1α) and the von Hippel-Lindau tumor suppressor, with strong potential to be used in monitoring immune cell therapies 57.

A recent preclinical study utilized PSMA as a reporter gene for noninvasive imaging of CAR T cells 58. PSMA is a cell surface protein whose expression is limited to the prostate gland making it an ideal reporter candidate as a reporter 59. A radiolabeled small molecule, 2-(3-(1-carboxy-5-[(6-[^18^F]fluoro-pyridine-3-carbonyl)-amino]-pentyl)-ureido)-pentanedioic acid (^18^F-DCFPyL), was developed as a highly sensitive and specific probe targeting PSMA 60. CAR T cells co-expressing anti-CD19 CAR and PSMA were detected with high sensitivity *in vitro* and *in vivo* while CAR T cell infiltration into primary and metastatic Nalm6 tumors was visualized 58. Interestingly, response to CAR T cell therapy was not correlated with detection of CAR T cells in the blood using flow cytometry, which is the current clinical practice. However, CAR T cell infiltration visualized by PET was correlated to response, indicating that noninvasive imaging of CAR T cells may be a more clinically relevant prognostic tool.

While endogenous biomarkers are typically attractive for imaging cancer, the sensitivity and/or specificity is often not sufficient to drive clinical adoption and success. Recently, macrophages were engineered as immunological sensors that generate a synthetic bioluminescent reporter when they polarize toward a more M2-like tumor-associated macrophage profile 61. This strategy was accomplished by pairing luciferase expression to the activation of the immunosuppressive arginase-1 promoter. In cancer models, once the macrophages trafficked to tumor sites, arginase-1 was activated, simultaneously releasing luciferase for detection by bioluminescence as a site-specific indicator of M2-polarization.

These preclinical studies illustrate the utility of *in vivo* labeling to track and monitor CAR T cells and macrophages. Both *ex vivo* and *in vivo* labeling approaches provide advantages including simplicity or temporal control, respectively, as well as drawbacks, such as a time-limited protocol or potential immunogenicity, respectively. However, *in vivo* imaging techniques using reporter genes provide the ideal characteristics for clinical practice due to the freedom to image at any timeframe after treatment. Reporter genes, such as PSMA and ^18^F-DCFPyL, allow monitoring of CAR T cell trafficking to and infiltration into the tumor and metastatic sites while providing better prognostic power compared to serial assessment in the peripheral blood. In conclusion, PET imaging of CAR T cells using reporter systems is highly translatable in the clinic and shows potential as a prognostic tool.

Another strategy of significant interest is imaging infiltration of tumors by T cells. Cytotoxic CD8^+^ T cells directly mediate the anti-tumor immune response and, thus, are an important target for noninvasive imaging 62. Similarly, CD4^+^ T cells play an essential role in shaping the immune landscape of the TME and visualizing their presence in the tumor holds prognostic value 63. Several strategies have been developed to image T cells in both preclinical and clinical settings. This includes using antibody and antibody fragments targeting CD3, CD4 and CD8, markers of T, helper T and cytotoxic T cells, respectively 64. A radiolabeled murine anti-CD3 antibody was found to be able to detect tumor infiltrating lymphocytes in a syngeneic murine model 72 hours post-injection 65. *In vivo* imaging using a ^89^Zr-radiolabeled anti-CD4 cys-diabody resulted in high uptake in lymphoid organs 20 h post-injection 66. ^89^Zr-radiolabeled minibodies specific for mouse CD8 developed for PET imaging of CD8^+^ T cells resulted in high uptake in the lymphoid organs in mice 4 hours post-injection 67. These preclinical studies demonstrate that *in vivo* imaging of T cells is feasible.

Other preclinical studies were able to correlate the T cell infiltration into tumors with treatment outcomes. A ^89^Zr-labeled anti-CD3 antibody used in a preclinical colorectal cancer model found that tumors that responded to anti-CTLA-4 treatment had higher T cell infiltration compared to non-responders 68. A cys-diabody against murine CD8 molecule was developed for detection of tumor-infiltrating CD8^+^ lymphocytes 69. In a murine model of colorectal cancer, mice were treated with an anti-PD-1 antibody and imaged with the radiolabeled cys-diabody 48 hours later. Animals that received the treatment were found to have greater infiltration of CD8^+^ T cells into the tumors, indicating the predictive power of CD8^+^ PET imaging in response to anti-PD-1 treatment 69. A nanobody against murine CD8 was developed and used to detect CD8^+^ T cells *in vivo*. Addition of a 20 kDa polyethylene glycol (PEG) moiety to the nanobody significantly enhanced signal-to-background ratio and reduced uptake in the kidneys, which was attributed to its increased circulatory half-life and hydrophilicity 70. Homogeneous distribution of CD8^+^ T cells in tumors was found to be a predictive marker of response to CTLA-4 blockade. In a B16 melanoma model, mice treated with CTLA-4 blockade and GVAX vaccination 71 were monitored longitudinally by CD8^+^ PET imaging once a week for four weeks. The ^89^Zr-labelled PEGylated anti-CD8 nanobody was similarly used to image T cell infiltration in a murine colorectal cancer model treated with PD-1 blockade 21. Responder animals showed a significant increase of T cell infiltration into the tumors, whereas T-cells in non-responders remained mostly around the tumor periphery 21. These studies illustrate that infiltration of T cells into tumors is a predictive response to immunotherapy and can easily be translated into the clinic.

While imaging of infiltrating T cells using anti-CD3 or anti-CD8 antibodies has prognostic value, detected T cells may be exhausted or anergic and not capable of actively mounting an anti-tumor response. An important attribute of activated T cells is their increased surface expression of costimulatory molecules such as ICOS, 4-1BB, and OX40 72-74. Therefore, noninvasively imaging these costimulatory molecules can provide a better indication of T cell activation. A recent study using a ^89^Zr-labeled anti-ICOS antibody in a lung cancer model detected activated CD4^+^ and CD8^+^ T cells in the tumor and tumor-draining lymph nodes in response to a STING immune agonist treatment 75. In a similar study, a radiolabeled anti-OX40 antibody was used to detect activated T cells in A20 B cell leukemia tumors and tumor draining lymph nodes 4, 16, and 24 hours after tracer administration in response to CpG vaccination 76. These results show that imaging activated CD8^+^ T cells is a valuable tool for assessing the immune response to cancer immunotherapy.

### Clinical imaging of T cells

The presence of activated cytotoxic CD8^+^ T cells are important for initiating and mediating a successful response to CTLA-4 and PD-1/PD-L1 checkpoint blockade treatments 77-79 (Figure 5) and has been shown to be of prognostic value for tumor response in patients 80. Imaging of CD8^+^ cells is, therefore, an attractive approach for assessment of the anti-tumor status of TME in patients.

^89^Zr-IAB22M2C, a radiolabeled 80 kDa anti-CD8 minibody, is currently being evaluated in a phase II multicenter study (NCT03802123). IAB22M2C is an engineered bivalent homodimer with each monomer consisting of a single-chain variable fragment (scFv) linked to the human IgG1 CH3 domain from the humanized heavy and light chain sequences of murine anti-human OKT8 antibody. The minibody is humanized, biologically inert, and does not interact with the FcRn recycling receptor 67, 69, 81, 82. Preclinical studies of IAB22M2C and desferrioxamine -conjugated (Df)-IAB22M2C showed retention of high-affinity binding to human T cells (binding EC50 = 0.4 nM) and HPBALL leukemia cells and no measurable impact on proliferation or depletion of CD8^+^ T cells with incubation with peripheral blood mononuclear cells (PBMCs) from healthy human donors 83. No acute effects of intravenous administration of Df-IAB22M2C were noted on CD8^+^ T cell populations or cytokine release in humanized NSG mice engrafted with CD34^+^ stem cells.

**Figure 8:**
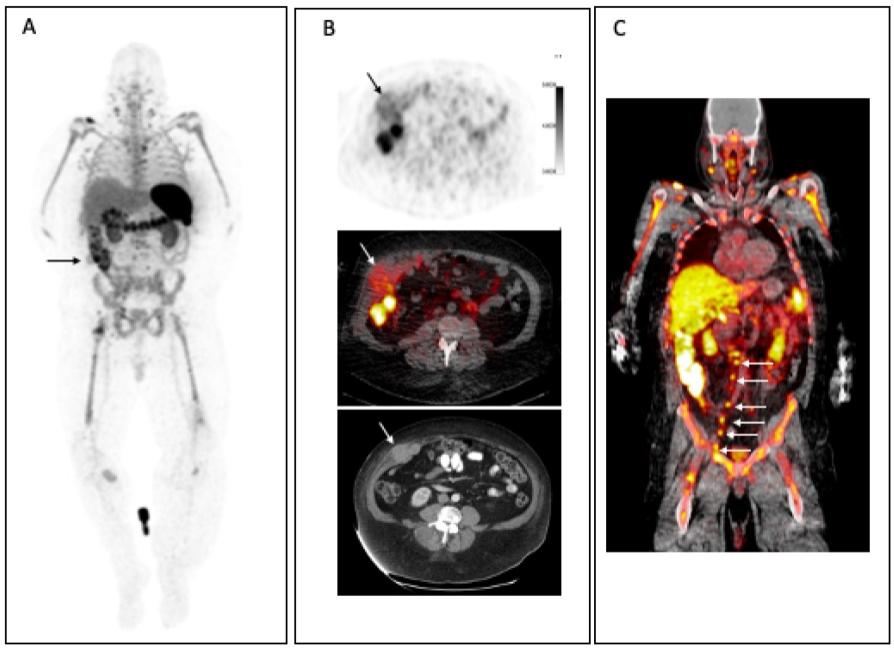
Patient with metastatic melanoma. **(A)** MIP images at 24h. p.i 3.0 mCi ^89^Zr-Df-IAB22M2C demonstrates prominent uptake in spleen, bone marrow and nodes, which are sites of T cell-rich tissue. Multiple focal areas of CD8 tracer uptake are noted including right flank muscle metastasis (arrow), lymph nodes in the neck, chest, pelvis, and groin and soft tissue in right lower extremity. **(B)** CD8+ tracer uptake is seen in the corresponding right flank muscle metastasis on axial CD8+ PET, fused CD8 PET/CT images and diagnostic CT. **(C)** Coronal PET/CT fusion images highlight the pelvic and retroperitoneal CD8+ nodal uptake (arrows).

A phase I first-in-human study with ^89^Zr-Df-IAB22M2C PET imaging showed specific targeting of CD8^+^ T cell-rich tissue in patients with multiple solid tumors that were either on treatment with checkpoint inhibitors or planned to be treated with checkpoint inhibitors 84. Prominent uptake in bone marrow, spleen, and lymph nodes at 6-24 h post-injection suggested selective accumulation in CD8^+^ T cell-rich tissues (Figure 8). ^89^Zr-IAB22M2C was noted in lesions in two patients that were being treated with immune checkpoint inhibitor therapy, suggesting possible enhanced modulation of TILs and presence of higher concentration of CD8^+^ T cells visualized by ^89^Zr-Df-IAB22M2C PET/CT imaging, which was confirmed by biopsy correlation that showed CD8^+^ T cell infiltration by immunohistochemistry in ^89^Zr-Df-IAB22M2C-positive lesion. In one patient who had not received immunotherapy, lesions were negative on ^89^Zr-IAB22M2C imaging, possibly suggesting a lack of TIL stimulation and infiltration. ^89^Zr-IAB22M2C-negative tumors in three patients with metastatic lung cancer lacked ^18^F-FDG uptake on concurrent PET imaging suggesting either lack of active disease or chronic low TIL infiltration 84. A phase II multicenter study evaluating ^89^Zr-IAB22M2C imaging at baseline prior to and after checkpoint blockade treatment on patients with multiple solid tumors is underway (NCT03802123). In this study, ^89^Zr-IAB22M2C imaging and uptake is to be correlated with biopsies. Another CD8-targeting antibody for PET imaging, ^89^Zr-ZED88082A, is being evaluated in a phase I trial (NCT04029181, NCT02478099).

Antibody or antibody fragments specific for CD3, a general marker for all T cells, and CD4, a marker for helper T cells, are being developed for clinical translation. ^89^Zr-labeled GK1.5 cDb is a radiolabeled diabody targeting CD4 that has shown T cell infiltration related uptake in spleen, blood, lymph nodes, and thymus 66. A novel approach uses bispecific T-cell engager ^89^Zr-AMG 211 directed against tumor carcinoembryonic antigen (CEA) and CD3 on T-cells. In a small clinical study in 9 patients with advanced gastrointestinal adenocarcinomas, imaging showed uptake in CD3-rich tissue such as spleen and bone marrow 85. Although these approaches are in the early phases of developments, they may allow for imaging and tracking of T cells in real time.

## Imaging the tumor stroma

### Imaging angiogenesis

Angiogenesis is the process by which new blood vessels are developed from a pre-existing vascular structure. This process can be induced by the tumor, due to the absence of oxygen and nutrients, and is important for tumor growth and metastasis. The regulation of angiogenesis involves many signaling pathways, receptors, and ligands such as αvβ3 integrin, platelet-derived growth factor (PDGF), matrix metalloproteinases (MMPs), hypoxia-inducible factor 1 (HIF-1), and vascular endothelial growth factor (VEGF). All these pathways are potential targets for therapies and can be considered as targets for imaging agents.

Considering these various angiogenesis-stimulating factors, VEGF is considered the most potent and predominant factor 86. VEGF-A is the most prominent growth factor in the VEGF family and it stimulates angiogenesis in healthy and tumor tissue by signaling through VEGF receptor-2 87. Proteolytic enzymes in tumors may activate or release growth factors from the extracellular matrix (ECM) or act directly on the ECM itself, thereby facilitating angiogenesis or tumor cell migration. Bevacizumab is an FDA-approved monoclonal antibody that targets VEGF-A and is approved for the treatment of multiple cancer types, including colorectal, lung, breast, brain, and ovarian cancer. It has also been utilized as a PET imaging agent both preclinically and clinically. A preclinical PET imaging study found much higher tumor uptake of ^89^Zr-bevacizumab compared to ^89^Zr-IgG in nude mice implanted with the human ovarian cell line, SKOV-3, after 72 hours 88. *Ex vivo* biodistribution studies after 168 hours showed high uptake of the radioisotope in the tumor, spleen, liver, and bone.

Additionally, a clinical PET imaging study conducted on renal cell carcinoma patients using ^89^Zr-bevacizumab as an imaging agent before and after treatment with bevacizumab with interferon-α or sunitinib found tumor uptake of the imaging agent was high yet heterogeneous in patients. Interestingly, treatment with bevacizumab in combination interferon-α strongly decreased tumor uptake of the imaging agent, indicating a correlation between response to treatment and tumor PET signal, whereas sunitinib treatment resulted in a modest decrease in uptake 89. Similarly, a PET clinical study using ^89^Zr-bevacizumab in children with diffuse intrinsic pontine glioma (DIPG) found five of seven patients displayed PET signal at the tumor site with one patient showing signal at multiple metastatic sites 90.

Although VEGF-A is an important growth factor in the angiogenic process, its cognate receptor, VEGFR-2, is a major mediator of angiogenesis. VEGFR-2 is highly expressed in a range of solid tumors 91 and is associated with ovarian cancer progression 92. Different approaches have been taken to image VEGFR-2, the earliest of which was the use of radiolabeled VEGF-A. A preclinical study utilizing the VEGF_165_ and VEGF_121_ growth factor, the active soluble secreted VEGF isoforms, radio iodinated with ^123^I found the radiolabeled agent can bind to human umbilical vein endothelial cells as well as a range of established tumor cell lines. This led to clinical studies utilizing ^123^I-VEGF_165_ and ^123^I-VEGF_121_ in patients with gastrointestinal tumors 93 and pancreatic lesions 94. These proof-of-principle studies led to the use of VEGF as a PET imaging agent for the detection of VEGFR-2 expression. Nude mice implanted with U87 glioblastoma cells subjected to PET imaging using ^64^Cu-DOTA-VEGF_121_ were found to have high uptake of the radiotracer in small (high VEGFR-2 expression) tumors but low uptake in larger (low VEGFR-2 expression) tumors 95. Unfortunately, high renal uptake with no observable clearance was also observed, likely due to high VEGFR-1 expression in the kidneys. To overcome this challenge, a VEGF_121_ mutant (VEGF_DEE_) was developed that is specific for VEGFR-2 only, thereby avoiding renal uptake and toxicity 96. The study found high uptake of ^64^Cu-DOTA-VEGF_DEE_ in VEGFR-2 expressing 4T1 tumors implanted in BALB/c but significantly lower renal uptake compared to ^64^Cu-DOTA-VEGF_121_. A more recent study utilized a 12-amino-acid peptide within exon 6 of VEGF-A (VEGF_125-136_) that was first identified as an inhibitor to VEGFR as a targeted PET imaging agent. They found ^64^Cu-DOTA-VEGF_125-136_ effectively targets B16F10 and U87 cells *in vivo* with great pharmacokinetic properties only showing noteworthy kidney and liver uptake in *ex vivo* biodistribution studies 97.

### Imaging of fibroblasts

**Figure 9:**
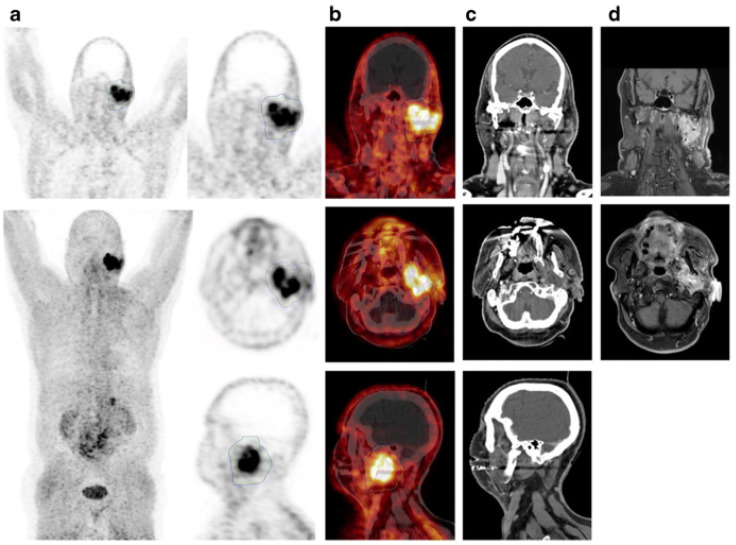
PET imaging with ^68^Ga-FAPI is superior to conventional MRI scan. **(A, B)** A patient receiving a PET scan with ^68^Ga-FAPI to noninvasively image a mucoepidermoid carcinoma lesion on the left parotid gland. PET images show tracer uptake at the tumor site with low background. **(C, D)** Conventional MRI imaging shows diffuse tumor infiltration, thereby making delineation of tumor and stromal tissue difficult and subjective. (Adapted for use under Creative Commons license http://creativecommons.org/licenses/by/4.0/ from Syed, M., Flechsig, P., Liermann, J., *et al.* Fibroblast activation protein inhibitor (FAPI) PET for diagnostics and advanced targeted radiotherapy in head and neck cancers. *Eur J Nucl Med Mol Imaging* **47,** 2836-2845 (2020). doi: 10.1007/s00259-020-04859-y)

Cancer-associated fibroblasts (CAF) are one of the main cellular components in the TME and play a major role in regulating the behavior of tumors. CAFs are activated fibroblasts with a mesenchymal cell lineage that secrete a variety of soluble factors that facilitate the progression of cancer stemness, immune regulation, angiogenesis, drug resistance, extracellular matrix remodeling, and other biological processes 98, 99, 100. Therefore, noninvasive imaging of CAFs can provide insight into the TME. Fibroblast activation protein (FAP) is a cell surface antigen of reactive tumor stromal fibroblasts found in epithelial cancers but not in normal tissues. More than 90% of lung, breast, and colon carcinomas have FAP expression in their stroma. Of note, FAP has been associated with poor prognosis. In patients with gastric cancer, upregulation of FAP was noted in poorly differentiated tumors and correlated with more adverse clinical and pathological characteristics including histology and pathological stage 101. In a phase I clinical study, a FAP-specific antibody, F-19, was developed and used as an imaging agent. The radiolabeled antibody was used for detection of FAP in the tumor stroma in patients with colorectal carcinoma where hepatic metastasis was shown 102. In another study, ^131^I-Sibrotuzumab, a humanized antibody against human FAP, was used for imaging in a phase I dose escalation study with cold Sibrotuzumab. Lesions were visualized with high contrast and no normal organ uptake was noted 103. Subsequent studies have focused on the development of FAP as a target for cancer therapies 104, 105. Furthermore, a ^68^Ga-radiolabeled FAP inhibitor ligand, ^68^Ga-FAPI (Figure 9), was shown to target tumor tissue in head and neck tumors with high signal-to-background ratio. Radiation planning volumes assessed with ^68^Ga-FAPI showed larger gross tumor volume as compared to conventional imaging 106. Further developments for targeting FAP include fluorescence activatable bispecific endoglin-fibroblast, FAP-targeting liposomes 107, and adoptively transferred CAR-T cells directed to FAP 108.

### Imaging cytokines

Although mutations in oncogenes and tumor suppressor genes are the drivers of tumor progression, the TME plays a pivotal role in tumor growth and dynamic between the tumor and immune cells. Cytokines in the TME can contribute to both an anti-tumor immune stimulatory (for example IL-2 family, INF-α, IL-10, IL-12) and pro-tumor inflammatory response (such as TNF- α, TGF-β, IL-8) 109. Cytokines present an anti-tumor response through increasing antigen-presentation, recruiting and enhancing the cytotoxicity of T cells 110. However, pro-tumor cytokines promotes an anti-inflammatory response that induces an immunosuppressive tumor landscape 111, 112. Therefore, the detection of cytokines in the TME can directly gauge the immune landscape of the tumor and provide a prognostic, and even predictive, tool for determining the response to immunotherapy.

Several preclinical studies have noninvasively imaged cytokines. For example, a ^89^Zr-labeled antibody has been developed to detect IFN-γ in a mammary cancer model 113. Mice implanted with breast cancer cells expressing HER2 when treated with a HER2-specific DNA vaccine were found to have higher uptake of the tracer in tumors. Interestingly, tracer uptake was positively correlated with response to therapy, indicating that IFN-γ can be a prognostic imaging marker 113. In another study, ^64^Cu was conjugated to Etanercept, a TNF Receptor 2 IgG1 Fc fusion protein that binds to and inhibits TNF-α, and used to noninvasively image TNF-α in a model of acute and chronic inflammation 114. Inflammation was induced by injection with 12-O-tetradecanoyl-phorbol-13-acetate (TPA) into the right ear and the tracer was subsequently injected. Tracer uptake significantly increased in the right ear after only a single injection of TPA, illustrating the sensitivity of Etanercept as an imaging agent.

Another PET imaging strategy involves the use of radiolabeled cytokines to detect activated T cells or other immune cells expressing their cognate receptors. Several studies have used radiolabeled Interleukin-2 (IL-2), a 15 kD alpha-helical protein produced by activated T cells that supports differentiation in regulatory T-cells and proliferation in effector T cells, for detection of activated T cells expressing the high affinity IL-2 receptor, comprised of a tricomplex IL2 receptor alpha (IL2RA) (CD25), beta (IL2RB) and common gamma chain 109, 115-118. A fluorinated IL-2, N-(4-[^18^F]fluorobenzoyl)-IL-2 (^18^F-FB-IL-2) has been used to detect activated T cells in the TME of a lung cancer model to monitor cancer therapy 119. Mice whose tumors were treated with either radiation or vaccination showed greater uptake of ^18^F-FB-IL-2 in their tumors, indicating an increased presence of activated T cells. Labeling of IL2 with (99m)Tc bifunctional chelating agent succinimidyl-6-hydrazinopyridine-3-carboxylate (HYNIC-NHS) and tricine, as co-ligand has been developed for imaging of IL-2 to track T cells in patients 120. It binds to the IL-2 tri- receptor complex, expressed on TILs, and following internalization, the Tc99m/18F moiety is imaged. Feasibility and safety assessment in patients was favorable with only minor grade I toxicities such as pain or pruritis noted. In clinical studies, ^99m^Tc-HYNIC-IL-2 imaging pre- and post-12 weeks of immune checkpoint therapy in metastatic melanoma showed correlation between change in lesion size post-therapy with change in the tracer uptake 121. A phase I clinical trial in melanoma patients treated with immune checkpoint inhibitors is underway (NCT02922283). ^18^F-labeled IL2 (N-(4-^18^F-fluorobenzoyl)interleukin-2 ^18^F-FB-IL2) has been evaluated preclinically 116 and, more recently, ^18^F-AlF-RESCA-IL2 and ^68^Ga-Ga-NODAGA-IL2 have also been developed and evaluated preclinically 122.

## Imaging using nanomaterials

**Figure 10:**
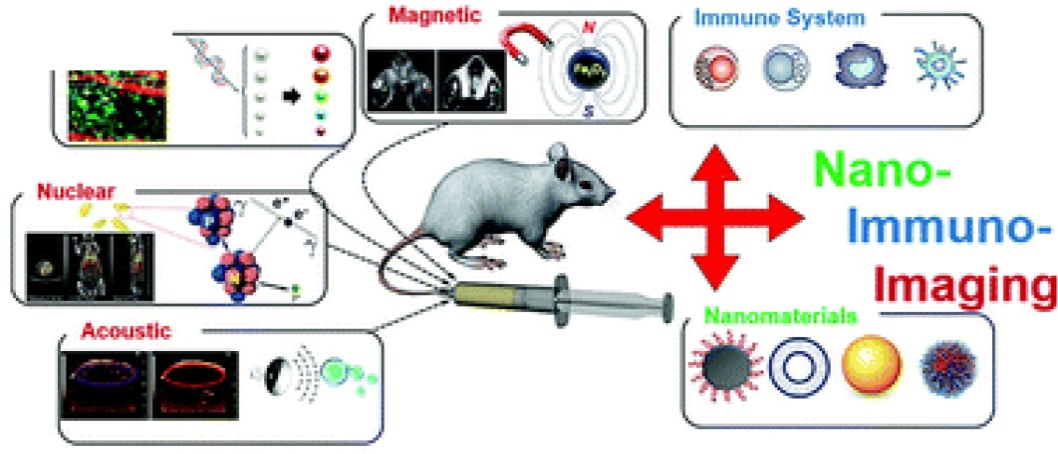
Modalities for nano-immunoimaging. (Adapted from Wang P et al. NanoImmunoimaging, Issue 4, 2020.)

Nanomaterials have been used as contrast agents for *in vivo* imaging for several decades due to their capacity for high payload and avidity, robust targeting, and multimodal functionality 123, 124. Contrast agent-containing nanomaterials moreover can be engineered to specifically bind/target immune cells and molecules in what is known as nano-immunoimaging, lying at the intersection of nanotechnology, imaging, and immunology 125, 126. Nano-immunoimaging spans the existing imaging modalities, including magnetic, optical, acoustic, and nuclear approaches 127 (Figure 10). Here we describe molecularly targeted nanomaterials and intrinsically targeted nanomaterials.

### Molecularly targeted nanomaterials

Nanomaterials are ideal to increase imaging agent avidity due to their high surface area and surface to volume ratios, allowing many targeting ligands per nanoparticle 123. Because tumor-associated macrophages (TAMs) can indicate cancer progression, the number of TAMs in a tissue is likely to impact immunotherapeutic response. A polyglucose nanoparticle (NP) was labeled with ^64^Cu to quantify TAMs in living subjects. Tumors high in TAM displayed >700% higher amounts of a model therapeutic NP versus TAM-deficient tumors based on PET, indicating that radiolabeled polyglucose NPs targeting TAM could be eventually used for patient stratification in immunotherapy trials 128. Many other ligands conjugated to NPs may be employed in order to target immune cells, include those typically used in immune targeting strategies for nuclear medicine as described elsewhere in this review, e.g., antibodies/nanobodies/ diabodies, peptides, small molecules, and nucleic acids 129.

### Intrinsically targeted nanomaterials

**Figure 11:**
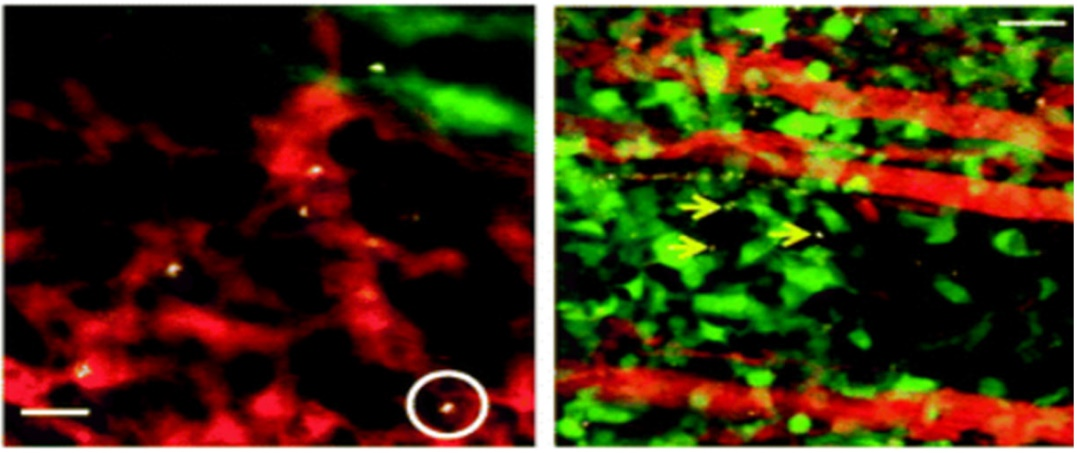
SWNT uptake into circulating cells and deposition into tumor in a mouse model of glioblastoma multiforme (u87-mg cells) (left). Representative intravial fluorescence image of single walled carbon nanotube (SWNT)-laden circulating cells in tumor vasculature (one example cell is circled). (Adapted from Wang P et al. NanoImmunoimaging, Issue 4, 2020; originally reproduced by permission of *Nature*.)

NPs can also display intrinsic cellular selectivity. We distinguish intrinsic selectivity from common properties, such as phagocytic uptake of many NP types by phagocytic cells (such as macrophages) that lead to uptake of many NP types. In contrast, NPs that are intrinsically selective will likely yield many benefits due to their capabilities to internalize in cell subsets other than macrophages, and to more simply inject the NPs *in vivo* without needing to harvest cells, isolate them, and then reinject them. In such *ex vivo* labeling cases, the cell phenotype could for instance change prior to reinjection, which adds risk as well as extra costs and time, making intrinsic *in vivo* labeling a highly attractive alternative to *ex vivo* labeling. The biggest challenge for *in vivo* labeling lies in endowing the capacity to target a particular subpopulation of cells with sufficient selectivity. This is true because most cell types must be identified by a set of multiple cell surface receptors (e.g., by flow cytometric analyses), not a single marker as would be required for typical *in vivo* targeting. While 100% targeting accuracy is not required to ensure effective delivery strategies in imaging (and therapy), it is clear that relatively high specificity is required alongside minimal off-target uptake. *In vivo*, exceptionally high intrinsic selectivity of single-walled carbon nanotubes (SWNTs) has been shown toward a subset of monocytes, inflammatory monocytes 130. Approximately 100% of circulating inflammatory (Ly-6C^hi^) murine monocytes in the blood circulating took up SWNTs after injection, while under 3% of any other circulating cell type, including other myeloid cell subsets such as other monocytes, took them up. Visualization of these processes using intravital microscopy (IVM) exposed exceedingly quick SWNT uptake into inflammatory monocytes in the blood and ensuing trafficking into the tumor as a 'Trojan Horse' (Figure 11) as shown by both IVM and photoacoustic imaging 125, 130. IVM is a powerful optical imaging tool that provides dynamic insights into biological and nanomaterial activities in living subjects at sub-micron resolution typically via fluorescence 131. Another key advantage of IVM is its capability for multi-plexing many different colors, at least 5 at a time, which could be used to separately label and simultaneously track multiple different immune cell types within the same animal. Indeed, while this strategy selectively targets monocytes, one of the major challenges in the field is to boost the number of immune cell subsets (including valuable targets like cytotoxic T cells, NK, and NKT cells) that NPs can very selectively internalize within, and this high-reward objective in nano-immunoimaging is particularly important to decrease side effects from off-target binding that may lead to extraneous and difficult-to-interpret image signal 125.

Optical coherence tomography (OCT) is an *in vivo* imaging modality that applies light scattering within tissues to generate images, typically contrast-free. It boasts high resolution (only slightly worse than fluorescence) and deeper penetration capabilities than IVM. While one disadvantage of OCT is that its contrast agents are far less developed than other modalities, recent progress has produced scattering nanomaterials that add the ability to molecularly target or otherwise track cells using imaging contrast agents 131. In one immune cell tracking application, macrophages and activated microglia in orthotopically implanted brain tumors naturally took up large gold nanorods, which produce substantial OCT contrast 132. Given the ability to dynamically track these myeloid cells within tumors down to single-cell resolutions, it may be possible to closely monitor intratumoral macrophage responses to chemo- and immunotherapies in the future.

We note that nanomaterials do not necessarily need to be targeted, intrinsically or molecularly, if internalization is achieved *ex vivo*. For instance, immune cell subsets that have been harvested can be specifically isolated, and then embedded with imageable nanomaterials *ex vivo*, intended for reinjection and homing to the tumor microenvironment for imaging 46, 133.

Photoacoustic imaging (PAI) is an emerging imaging modality that benefits from many of the advantages of optical and ultrasonic imaging by sending sound 'in' (via laser) and creating tomographic images from sound 'out' (via ultrasonic transducers) to produce high spatial resolution images in fairly deep tissues (up to 7-8 cm) 123. T cells labelled *ex vivo* with a photoacoustic dye were injected IV into mice bearing tumors. PAI was used as a cell tracking modality, able to assess T cell migration into tumors, with a peak at 12 hours at a depth of nearly 1 cm. Improvements to PAI immunoimaging approaches are likely, including development of PAI-active nanomaterial agents that target immune cells *in vivo*. For instance, SWNTs were used to target inflammatory monocytes to image inflammatory disease (atherosclerotic plaque) using PAI, and similar approaches are likely to enable PAI of cancer with utility in tracking the trafficking of inflammatory monocytes and macrophages in the TME 134.

## Conclusion

As novel molecularly targeted therapeutic agents are becoming available for different malignancies, predicting the response and pharmacodynamic assessment of therapy is becoming more critical, particularly for immunotherapy. Assessing tumor target expression for treatment planning and management remains a clinical challenge. The complex nature of the TME and the processes that independently or interactively act in response to the changes with treatment and tumor growth require sophisticated and accurate methods to evaluate these changes. Several approaches, as mentioned in this review, are being explored to address these challenges. However, such methods are in the early phases of development, either clinically or preclinically. While many of these approaches are unique and hold promise for enabling a better understanding of changes in the tumor milieu, further work is needed for successful translation and implementation in clinical care.

## Figures and Tables

**Table 1:** Radiolabeled molecules or peptides for imaging

Agent	Target	Class	Stage	Active clinical trials	References
^18^F- AraG	Deoxyguanosine kinase (dGK)	Small Molecule	Clinical	NCT04052412, NCT03142204, NCT04186988, NCT03129061, NCT03684655, NCT03367962	10, 11, 135
^18^F-CFA	dCK	Small Molecule	Clinical	NCT03409419	136
1-L-^18^F-FETrp	IDO	Small Molecule	Preclinical		137
					
					
^18^F-FAC	Deoxycytidine kinase (dCK)	Small Molecule	Clinical		8
^68^Ga-WL12	PD-L1 PD-L1 PD-L1 PD-L1 PD-L1 PD-L1	Peptide	Preclinical		138
^18^F-FPy-WL12		Peptide	Preclinical		24
^18^F-NOTA-Z_PD-L1_1_		Affibody	Preclinical		139
^64^Cu-DOTA-FN3_hPD-L1_		Adnectin	Preclinical		140
^68^Ga-NOTA-Nb109		Nanobody	Preclinical		141
^64^Cu-DOTA-HAC-PD1		High-affinity PD-1 ectodomain	Preclinical		142, 143

**Table 2:** Radiolabeled antibodies/antibody fragments/ligands for imaging

Agent	Target	Type	Stage	Clinical trials	References
^89^ Zr-Df-IAB22M2C	CD8	Minibody	Clinical	NCT03107663 (completed) NCT03802123	84
^64^Cu-NOTA-ipilimumab-F(ab')2	CTLA-4	F(ab')_2_	Preclinical		144
^64^Cu-NOTA-ipilimumab		Antibody IgG1	Preclinical		144
^64^Cu-DOTA-ipilimumab		Antibody IgG1	Preclinical		145
^89^Zr-ipilimumab		Antibody IgG1	Clinical	NCT03313323, 2012-003616-31	
^18^F-FB-anti-MMR 3.49	Macrophage Mannose Receptor (MMR)	Nanobody	Preclinical		19
^64^Cu- VHH4	MHC II	Nanobody	Preclinical		22
^64^Cu-NOTA-avelumab Fab	PD-L1	Fab	Preclinical		146
^111^In-labeled atezolizumab		Antibody IgG1	Preclinical		147
^64^Cu-DOTA-atezolizumab		Antibody IgG1	Preclinical		147, 148
^111^In-PD-L1.3.1		Antibody IgG1	Preclinical		149
^89^Zr-DFO-C4		Antibody IgG1	Preclinical		150
^64^Cu-WL12		Peptide	Clinical	NCT04304066	151, 152
^99m^Tc-NM-01		Nanobody	Clinical	NCT02978196 (completed)	
^89^Zr-envafolimab		Nanobody Fc fusion	Clinical	NCT03638804	153, 154
^18^F-BMS-986192		Adnectin	Clinical	2015-004760-11(completed) NCT03520634, NCT03843515, NCT03564197, NCT03843515, 2018-002643-28,	27, 155
^89^Zr-durvalumab		Antibody IgG1	Clinical	2015-005765-23, NCT03829007, NCT03853187	34
^89^Zr-labeled avelumab		Antibody IgG1	Clinical	NCT03514719	156
^89^Zr-labeled atezolizumab		Antibody IgG1	Clinical	NCT03850028, NCT04006522, NCT04222426, NCT02478099, 2019-001197-28, 2017-003511-20	32
^89^Zr-CX-072		Pro-antibody	Clinical	2016-002490-36	157
^64^Cu-pembrolizumab	PD-1	Antibody IgG4	Preclinical		158, 159
^89^Zr-pembrolizumab		Antibody IgG4	Clinical	NCT02760225, NCT03065764, NCT03446911*, 2015-004260-10, 2016-003819-36	158, 29
^89^Zr-nivolumab		Antibody IgG4	Clinical		27, 30
^89^Zr-DFO-AN-18	IFN - γ	Intact antibody	Preclinical		113
^64^Cu-DOTA-etanercept	TNF-α	Anti-TNF-α drug (Etanercept)	Preclinical		114
^18^F-FB-IL-2	IL-2 receptor	Labeled cytokine (IL-2)	Clinical	NCT03304223, NCT04163094, NCT02478099	117
^68^Ga-Ga-NODAGA-IL2		Labeled cytokine (IL-2)	Preclinical		122
^18^F-AlF-RESCA-IL2)		Labeled cytokine (IL-2)	Preclinical		122 160, 161
^89^Zr-Bevacizumab	VEGF	Intact antibody	Clinical	NCT01081613, NCT01338090, NCT01894451, NCT01028638, NCT00991978, NCT00831857, NCT00970970	

*PET imaging using radiotracer is considered a secondary outcome

**Table 3:** Reporter gene imaging T-cells

Agent	Reporter gene	Species	Type	Stage	
^18^F-FHBG	Herpes Simplex Virus - Thymidine Kinase (HSV-TK)	Herpes Simplex Virus 1	Enzyme	Clinical	162, 163
^18^F-DCFPyL	Glutamate carboxypeptidase 2 (PSMA)	Human	Cell surface enzyme	Preclinical	58
^99m^TcO_4_^-^	Sodium Iodine Symporter (NIS)	Human	Transporter	Preclinical	49
^123^I-MIBG, ^124^I-MIBG	Norepinephrine Transporter (NET)	Human	Cell surface receptor	Preclinical	50
^68^Ga-DOTATOC	Somatostatin Receptor 2 (SSTR2)	Human	Cell surface receptor	Preclinical	51
^18^F-TMP	Dihydrofolate reductase enzyme (DHFR)	Escherichia coli	Enzyme	Preclinical	164
